# Post-concussion symptoms and chronic pain after mild traumatic brain injury are modulated by multiple locus effect in the *BDNF* gene through the expression of antisense: A pilot prospective control study

**DOI:** 10.1080/24740527.2017.1362942

**Published:** 2017-09-13

**Authors:** Samar Khoury, Julia Segal, Marc Parisien, Anne Noreau, Patrick Dion, Rodrigo Benavides, Jean-François Giguère, Ronald Denis, Inna Belfer, Luda Diatchenko, Guy A. Rouleau, Gilles J. Lavigne

**Affiliations:** a Centre for Advanced Research in Sleep Medicine, Hôpital du Sacré-Cœur and Université de Montréal, Montréal, QC, Canada; b Department of Surgery, Hôpital du Sacré-Cœur and Université de Montréal, Montréal, QC, Canada; c The Alan Edwards Centre for Research on Pain, McGill University, Montréal, QC, Canada; d Montreal Neurological Institute and Hospital, Department of Neurology and Neurosurgery, McGill University, Montréal, QC, Canada

**Keywords:** Anti-sense RNA, brain-derived neurotrophic factor, chronic pain, mild traumatic brain injury

## Abstract

**Background**: Mild traumatic brain injury (mTBI) often results in post-concussion symptoms, chronic pain, and sleepiness. Genetic factors are thought to play an important role in poor prognosis.

**Aims**: The aims of this study are to (1) document the prevalence of pain and post-concussion symptoms in mTBI patients in acute and chronic phases (2) determine whether candidate genes predispose to post-concussive symptoms and pain.

**Methods**: Posttraumatic symptoms, evaluated using the Rivermead Post-Concussion Symptoms Questionnaire, and pain were assessed in 94 mTBI patients in the acute phase as well as in 22 healthy controls. Assessment was repeated in 36 patients after one year who agreed to participate in the follow-up visit. Gene polymorphisms and expression were assessed in mTBI patients and healthy controls.

**Results**: In the acute phase, mTBI patients with pain (69%) presented more psychological symptoms and sleepiness and were less able to return to work than those without pain. At one year, 19% of mTBI patients had persistent pain and psychological distress. Two haplotypes (H2 and H3) in the brain-derived neurotrophic factor (*BDNF*) gene were shown to be respectively deleterious and protective against post-concussion symptoms and pain in both acute and chronic phases. Protective haplotype H3 was associated with a decreased expression of the anti-sense of *BDNF* (*BDNF-AS*). Deleterious haplotype H2 predicted the development of chronic pain at one year, whereas H3 was protective.

**Conclusions**: This pilot study suggests a protective mechanism of a multilocus effect in *BDNF*, through *BDNF-AS*, against post-concussion symptoms and pain in the acute phase and possibly chronic pain at one year post-mTBI. The role of antisense RNA should be validated in larger cohorts.

## RÉSUMÉ

**Contexte:** Le traumatisme cranio-cérébral léger (TCCL) donne souvent lieu à des symptômes post-commotionnels, de la douleur chronique et de la somnolence. On croit que des facteurs génétiques jouent un rôle important dans le pronostic défavorable.

**Buts:** Les buts de cette étude sont : 1) documenter la prévalence de la douleur et des symptômes post-commotionnels chez les patients ayant subi un TCCL, au cours des phases aigue et chronique; ii) déterminer si des gènes du candidat le prédisposent à la douleur et aux symptômes post-commotionnels.

**Méthodes:** La douleur et les symptômes post-traumatiques, évalués à l’aide du questionnaire Rivermead sur les symptômes post-commotionnels, ont été évalués chez 94 patients ayant subi un TCCL au cours de la phase aigue, ainsi que chez 22 sujets témoins. Après un an, 36 patients qui avaient accepté de participer à une visite de suivi ont à nouveau été évalués. Les polymorphismes et l’expression des gènes ont été évalués chez les patients ayant subi un TCCL et chez les sujets témoins.

**Résultats:** Au cours de la phase aigue, les patients ayant subi un TCCL avec douleur (69 %) présentaient davantage de symptômes psychologiques et de somnolence et étaient moins aptes à retourner au travail que les patients sans douleur. Après un an, 19 % des patients ayant subi un TCCL souffraient d’une douleur persistante et de détresse psychologique. Deux haplotypes (H2 et H3) dans le gène BDNF se sont montrés respectivement délétère et protecteur contre les symptômes post-commotionnels et contre la douleur, tant au cours de la phase aigue que de la phase chronique. Le haplotype protecteur H3 a été associé à une diminution de l’expression de l’anti-sens de *BDNF* (*BDNF-AS*). Le haplotype délétère H2 a prédit le développement de la douleur chronique un an plus tard, tandis que H3 a été protecteur.

**Conclusions:** Cette étude pilote suggère l’existence d’un mécanisme protecteur d’un effet multilocus dans *BDNF* à travers *BDNF-AS*, contre les symptômes post-commotionnels et la douleur au cours de la phase aigue et possiblement au cours de la phase chronique, un an après le TCCL. Le rôle de la RNA anti-sens devrait être validé avec de plus grandes cohortes.

## Introduction

Mild traumatic brain injury (mTBI) refers to a blunt physical trauma to the head that is usually the result of a motor vehicle accident, fall, or sports-related injury. The annual incidence in North America is 100–300/100, 000.^1^ Historically, mild brain injuries were considered a concussion that would heal in a relatively short time. More accurately, it is referred to as a “silent epidemic” because it often leaves patients with post-concussion symptoms such as headaches, mood disturbances, deteriorated quality of life, sleepiness, and chronic pain.^2,3^

Post-concussion symptoms commonly co-occur. For instance, 42% of war veterans with traumatic brain injury (TBI) have chronic pain, posttraumatic stress disorder, and post-concussion symptoms.^4^ A systematic review across 12 studies reported headache prevalence at 58% and chronic pain at 51% in civilians (higher in mTBI, at 75%) and 43% in veterans.^5^ We published that mTBI patients with pain had more symptoms of depression and anxiety, higher pain catastrophizing scores, and worse sleep quality with persistent wake-type electroencephalographic activity during sleep than mTBI patients without pain.^6^ In the TBI sleep literature, sleep–wake disturbances, characterized by excessive daytime sleepiness, fatigue, and pleiosomnia, are the most common complaints from mTBI patients in the days following mTBI.^7–9^

Poor sleep complaints in mTBI patients are comorbid with pain in 20%–25% of cases. More specifically, sleepiness, related fatigue, and diurnal impairment are the most common complaints in this population.^6,10^ The exact mechanisms are still unknown, but it is speculated that hyperarousal mechanisms and central sensitivity might contribute to the co-occurrence of sleep disturbance and pain following injury.^8,11^

The clinical course and long-term prognosis of mTBI patients differs among individuals. Psychological factors, age, gender, and neurodegenerative factors were previously shown to predispose to the chronicity of symptoms; however, these factors alone do not account for all cases of chronicity.^12–15^

Another possibility is that mTBI may be a potential catalyst of chronic pain and that the presence of initial post-concussion symptoms (i.e., headaches, fatigue, memory loss) may influence recovery, making it essential to characterize pain contribution as a risk factor.^8,16,17^

Genetic predisposition has been proposed to explain part of the variability in individual outcomes following mTBI.^18–20^ Previous studies found that neurocognitive performance following trauma and severity of concussion have been associated with a variety of polymorphisms, with the most commonly identified in apolipoprotein E (ApoE), interleukin-6 (IL-6), catechol-O-methyltransferase (COMT), dopamine receptor D2 *DRD2/Ankyrin Repeat and Kinase Domain Containing 1 (ANKK1)* Taq1A polymorphism and the brain-derived neurotrophic factor (BDNF), with conflicting results.^21–26^ The *BDNF* gene is located on chromosome 11p14, is 67 kbp long, and spans 12 exons. The expression of BDNF is highly regulated at the level of transcription.^27^ The Val66Met polymorphism (rs6265), which results in a valine-for-methionine substitution at codon 66, was reported to reduce intracellular trafficking and activity-dependent secretion of BDNF.^28^ The anti-sense BDNF (*BDNF-AS*) is known to inhibit BDNF mRNA expression, possibly through RNA duplexes, but changes in the expression of *BDNF-AS* have not been associated to date with differences in TBI outcomes.^27,29^ Neurotrophins, especially BDNF, play an important role in both the modulation of pain-related pathways and synaptic plasticity.^30,31^ Its most well-known function is to modulate synaptic efficacy, which results in long-term potentiation, dendritic growth, and synaptic formation and stabilization.^32,33^ In a recently published study, BDNF delivered into the brain of mice following experimentally induced TBI improved neurological and cognitive functions, thereby providing a neuroprotective effect.^34^ BDNF might therefore have an important role in synaptic plasticity of the nociceptive pathway following mTBI through decreased expression of its anti-sense.

Given the large number of mTBI patients seen in emergency departments and the cost for society of managing post-concussion symptoms, it is critical to associate factors and identify early predictors of chronic symptoms in order to facilitate screening for early intervention.

The objectives of this study are first to identify the contribution of pain in post-concussion symptoms by comparing mTBI patients with pain to mTBI patients without pain. The second objective is to verify whether known polymorphisms in previously studied genes predispose to general post-concussive symptoms and more specifically to pain following acute mTBI in this cohort and to assess their related gene expression. The third objective is to assess prospectively whether genetic factors predispose for the transition to chronic pain one year post-mTBI. The hypothesis is that functional polymorphisms are important predictors of poor prognosis and chronic pain following mTBI.

## Materials and methods

### Subjects

The study was approved by the ethics committee of the Sacré-Coeur hospital (where subjects were recruited) and the Centre Hospitalier de l’Université de Montréal (where blood samples were stored and analyzed). All patients gave informed consent before data and blood were collected. In a prospective case–control study design, data were collected from 102 mTBI patients and 22 healthy control subjects with no history of TBI.

The presence of mTBI was ascertained within 6 weeks of injury and diagnosis was confirmed by a trauma neurosurgeon (JFG) based on the 2004 World Health Organization (WHO) Task Force criteria^35^: 13–15 on the Glasgow Coma Scale 30 min postinjury; loss of consciousness for 30 min or less; posttraumatic amnesia for less than 24 h; and age 18–65 years. Patients were excluded if they had gross cognitive or speech dysfunction; use of any psychotropic medications or other drugs known to influence pain perception; history of chronic pain or fibromyalgia before mTBI; or presence of major neurological or psychiatric disorders. Eight patients were excluded for the following reasons: not meeting the WHO task force inclusion criteria (*n* = 2), major depression (*n* = 3), leukemia (*n* = 1), chronic alcohol use (*n* = 1), and collagenosis (*n* = 1). The final sample included 94 mTBI patients and 22 healthy control subjects.

All subjects completed a series of questionnaires and performed the Psychomotor Vigilance Test (PVT) as described below, and all had blood drawn for genotyping. Cell lines were cultured for gene expression analysis from mTBI patients and healthy controls.

Patients were classified as mTBI patients with pain (*n* = 65; 69%) if they reported pain during the interview and scored higher than “mild pain” on question 7 of the Medical Outcome Study Short-Form 36 (SF-36): “How much bodily pains have you had during the past 4 weeks?” Posttraumatic headache was also assessed, defined as a secondary headache that develops within 7 days after head trauma or after regaining consciousness.^36^ The other 29 mTBI patients were therefore classified as mTBI without pain.

At one year posttrauma, all mTBI patients were invited to participate in a follow-up protocol and 36 (38%) agreed. The same experimental paradigm was repeated except for genotyping and cell culture.

### Questionnaires

Medical diagnoses and reports were used to confirm patient eligibility. General physical and emotional health questionnaires, translated in the French language and listed below, were administered at two time points: 6 weeks and one year posttrauma. Data on demographics, education, and return to work were also compiled.

#### Pain-related questionnaires

Bodily pain intensity was determined on a 100-mm visual analogue scale (VAS).

##### Pain Catastrophizing Scale

The Pain Catastrophizing Scale is a validated tool that measures catastrophic pain-related thinking. It assesses the three components of catastrophizing: rumination, magnification, and helplessness, using 13 questions rated on a scale of 0 to 4. Higher scores indicate greater catastrophizing.^37^

##### Migraine Disability Assessment

The Migraine Disability Assessment (MIDAS) is a seven-item questionnaire. The first five items assess the influence of headaches over the last 3 months. The last two questions assess the total number of days with migraine attacks and the mean pain intensity. The MIDAS score is calculated using the first five questions and the number of days in which migraines interfered with these activities. Disability scores were classified as follows: minimal (0–5), mild (6–10), moderate (11–20), and severe (>21).^38^

#### Post-concussion symptoms, psychological questionnaires, and quality of life

##### Rivermead post-concussion symptoms

Post-concussive symptoms were assessed using the Rivermead Post-Concussion Symptoms Questionnaire in the acute phase posttrauma (<6 weeks). This is the most widely used validated questionnaire for post-concussive symptoms. It contains 16 items that measure the presence and severity of cognitive, emotional, and somatic complaints on a five-point scale (1 = *no problem* to 5 = *severe problem*). The items are headaches, dizziness, nausea, noise and light sensitivity, sleep disturbance, fatigue, irritability, depressive symptoms, frustration, memory, concentration, taking longer to think, blurred and double vision, and restlessness. The overall score is obtained by summing all scores. Higher scores indicate more severe post-concussion symptoms.^39^

##### SF-36

To assess quality of life after mTBI, all patients completed the standard SF-36, a validated 36-item questionnaire divided into eight scales. For the interest of this study, we will focus on the global SF-36 score and the bodily pain SF-36 scale only. Higher scores indicate better quality of life. Scores for the eight scales range from 0 to 100.^40^

##### Beck Depression Inventory–II and the Beck Anxiety Inventory

These self-administered questionnaires contain 21 items that assess depression and anxiety on a score from 0 to 3. Higher total scores indicate more severe depressive and anxiety symptoms. Scores are classified as minimal, mild, moderate, and severe.^41,42^

##### Impact of Event Scale–Revised

This self-rated questionnaire assesses posttraumatic stress disorder symptoms.

Twenty-two questions are addressed: eight questions measure intrusion symptoms, eight measure avoidance symptoms, and six measure hyperarousal symptoms. Patients rate their perceived severity of posttraumatic stress disorder symptoms on a five-point scale (0–4). Scores above 26 are classified as severe.^43^

#### Sleep–wake disturbance questionnaires

##### Psychomotor vigilance testing

Psychomotor vigilance testing (PVT-192; Ambulatory Monitoring Inc., Ardsley, NY) was used to assess sleepiness and reaction time.^44^ Subjects were given a handheld computerized device with a red light-emitting diode display of a three-digit millisecond counter and were instructed to press a response button as soon as a visual stimulus appeared on the screen during a 10-min period. Subjects were given pretest training to minimize the practice effect. Mean reaction time (RT) was collected in milliseconds as the primary variable. Higher mean RT represents more sleepiness.

##### The Stanford Sleepiness Scale and fatigue assessment

The Stanford Sleepiness Scale is a one-item question that assesses levels of sleepiness. Its seven-point Likert-type descriptors range from 1 = *feeling alert* to 7 = *no longer fighting sleep*.^45^ Fatigue was assessed by asking: “Please select your current degree of energy.” Possible answers on seven-point Likert-type items ranged from 1 = *full energy* to 7 = *physically exhausted*.

### Candidate gene identification and genotyping

Genomic DNA was extracted from blood using the Puregene DNA kit (Gentra System, USA) according to the manufacturer’s protocol.

From the literature cited in Table 1, a list of candidate genes was identified as being potentially relevant to traumatic brain injury. This list includes apolipoprotein E (ApoE); ApoE promoter; poly[ADP ribose] polymerase 1 (PARP-1); the interleukins IL-1a, IL-1b, IL-6; catechol O-methyl transferase (COMT); p53; dopamine receptor D2/ANKK1 (DRD2 Taq1A polymorphism); brain-derived neurotrophic factor (BDNF); neurofilament heavy polypeptide (NEFH); and neuroglobin (NGB).^22,23,25,46–54^ Using Hap Map Genome Browser release #28 based on data available in August 2010, single nucleotide polymorphisms (SNPs) and tagSNPs were identified and were retained if they had a minor allelic frequency of at least 5% in the EUR (European Ancestry) population. Genotyping of all SNPs was performed using Sequenom IPLEX Gold Technology (San Diego, CA), according to manufacturer’s instructions, at Génome Québec Innovation Center, Canada.

**Table 1.** Details of candidate SNPs selected from the literature.

Genes	SNP	Chromosome	Location	MAF (%)	Phenotype	Reference
ApoE	rs429358	19	45411941	11.4	Neuropsychological assessment	46
	rs7412	19	45412079	8.6		
ApoE promoter	rs405509	19	45408836	47.2	Concussion in athletes	47
	rs449647	19	45408564	18.1		
PARP-1	rs1109032	1	226561403	17	Glasgow Coma Scale outcome	48
	rs3219090	1	226564691	31.4		
	rs3219119	1	226556443	31.5		
	rs2271347	1	226549498	23.6		
IL-1a	rs1800587	2	113542960	27.8	Glasgow Coma Scale outcome	49
IL-1b	rs1143634	2	113590390	22.4	Glasgow Coma Scale outcome	50
	rs16944	2	113594867	31.6		
IL-6	rs1800795	7	22766645	41.6	Brain injury severity	22
COMT	rs4680	22	19951271	49.9	Executive functions	23
p53	rs1042522	17	7579472	22.9	Glasgow Coma Scale outcome	51
*DRD2*/*ANKK1*	rs1800497	11	113270828	18.2	Cognitive outcome	25
BDNF	rs1519480	11	27675712	31.1	Memory and processing speed	52
	rs7124442	11	27677041	30		
	rs6265	11	27679916	20.9		
	rs11030101	11	27680744	45.9		
	rs11030102	11	27681596	23.1		
	rs11030104	11	27684517	21.7		
	rs11030107	11	27694835	23.1		
	rs7103411	11	27700125	21.3		
	rs7127507	11	27714884	30		
	rs7934165	11	27731983	47.4		
	rs11030121	11	27736207	31.7		
	rs12273363	11	27744859	20.3		
	rs908867	11	27745764	8.4		
NEFH	rs165602	22	29886043	13.7	Concussion in athletes	53
	rs3815335	22	29881468	30		
NGB	rs3783988	14	77734580	22.5	Animal model of severe pathology	54

SNP = Single nucleotide polymorphism; MAF = minor allelic frequency; ApoE = apolipoprotein E; PARP-1 = poly[ADP ribose] polymerase 1; IL = interleukin; COMT = catechol-O-methyltransferase; DRD2 = dopamine receptor D2; ANKK1 = BDNF = brain-derived neurotrophic factor; NEFH = neurofilament heavy polypeptide; NGB = neuroglobin.

### Cell line culture and RNA extraction

Lymphoblastoid cell lines were derived using standard methods from whole blood of mTBI patients and healthy controls and stored in liquid nitrogen until further use. Cells were subsequently thawed and resuspended in 10 ml of B cell media (Iscove’s Modified Dulbecco’s Medium, 20% fetal bovine serum, 1% penicillin streptomycin, 1% l-glutamine). RNA was extracted from approximately one million lymphoblastoid cells using the AllPrep DNA/RNA Mini kit (QIAGEN, Hilden, Germany) according to the manufacturer’s protocol. The samples were treated with DNase to eliminate genomic DNA contamination. RNA (10 µl) was then reverse transcribed for a total volume of 20 µl containing 10× RT Buffer, 25× dNTP mix (100 mM), 10× Random Primers, Multiscribe Reverse Transcriptase, and RNase inhibitor (High Capacity cDNA Reverse Transcription Kit, Applied Biosystems, USA).

### Expression quantitative trait loci

A database search for statistically significant SNPs in expression quantitative trait loci (eQTL) was done using the publicly available database Genotype–Tissue Expression project.^55^ The Genotype–Tissue Expression project provides correlations between genotype and a tissue-specific gene expression. For the purpose of this study, we extracted public gene expression data for the following selected tissues: human brain cortex (main sampling site: the right cerebral frontal cortex) and peripheral nerve (main sampling site: the left tibial nerve).

Due to the importance of dorsal root ganglia (DRG) in pain processes, we also looked for the DRG eQTLs data set obtained from the Diatchenko lab (diatchenko.lab.mcgill.ca:/DRG-eQTLs/).^56^ In short, the cohort includes samples that were collected postmortem from brain dead subjects following asystole. They consist of snap-frozen DRG sourced from bilateral lumbar L4 and L5. Samples are courtesy of the CORE/CORID repository at the University of Pittsburgh. A total of *N* = 214 samples with matched genotype/RNA levels were used in this study. The cohort composition was as follows: median age 52, 199/214 Caucasian, 105 females and 109 males.

Genes whose expression was shown to be modulated by statistically significant SNPs in both databases were tested using quantitative polymerase chain reaction (qPCR).

The qPCR reaction was carried out in the ProFlex PCR System (Applied Biosystems) for 10 min at 25°C, 120 min at 37°C, and 5 min at 85°C, followed by cooling to 4°C using cDNA obtained from DNAse-treated RNA samples. Amplifications were performed using TaqMan probe (Hs02718934_s1 and Hs01010223_m1) whose primers map to *BDNF* and *BDNF-AS*, respectively. Assays were performed in triplicates and results were repeated in a second round and compared to endogenous levels of glyceraldehyde 3-phosphate dehydrogenase (GAPDH).

### Statistical analysis

Statistical analyses were performed using SPSS (PASW 18, IBM Corporation, USA). Data are reported as mean ± SD unless otherwise specified. Group comparisons were performed using a Student’s *t* test (two groups, normal distribution), one-way analysis of variance (ANOVA; three groups), or a Mann-Whitney U test when data were not normally distributed and a chi-square test in categorical data cases. A repeated measure ANOVA was used to compare changes across repeated tests (questionnaire scores, PVT) at the two time points.

Genetic association analysis was performed using PLINK version 1.07-DOS (http://pngu.mgh.harvard.edu/~purcell/PLINK/).^57^ Single SNP *P*-values were obtained using association test with the number of minor alleles. A spectral decomposition matrix was applied to account for multiple testing and significance was established at *P* ≤ 0.002^58^ An association between SNPs and all available phenotypes was performed.

Haploview 4.2 software (Cambridge, MA, USA) was used to construct linkage disequilibrium (LD) blocks with the solid spine method criteria. SNPs within a block where then used for the generation of haplotypes. Haplotype analysis was performed using the expectation–maximization algorithm adapted by Zaykin et al. where haplotype frequencies and associations were generate per individual^59^ in a fixed set of marker mode. Only haplotypes with more than 10% frequency were used. This method is advantageous because it considers data from multiple correlated markers to given phenotypes.

## Results

Ninety-four mTBI patients (67 males, 27 females; mean age 37.7 ± 12.7 years) were assessed 46.1 ± 22.6 days posttrauma. Traumatic brain injury was the consequence of a motor vehicle accident for 34 patients (36.1%), a fall for 26 (27.7%), a cycling accident for 19 (20.2%), an assault for 11 (11.7%), and a pedestrian hit by a motor vehicle for four (4.3%). Sixty-five patients (69%) reported pain in the acute posttrauma phase and were classified as mTBI patients with pain. The most common type of pain was accident-related head and neck as well as back pain. Patients with lower Glasgow Coma Scale scores (13 and 14) are protected against pain and an initial Glasgow Coma Scale score of 15 report more pain at 6 weeks (odds ratio [OR] = 0.18; 95% confidence interval [CI], 0.04, 0.73; *P* = 0.016).

### Pain and psychological assessment

Although none of the mTBI patients reported chronic pain prior to traumatic brain injury, 21 (22.3%) reported occasional pain, namely, back pain (11), neck pain (4), headaches or musculoskeletal pain or shoulder pain (3), knee pain (2), abdominal pain (2), and foot pain (1). In all patients, except for abdominal pain and headaches, the occasional pain was related to work posture or sports. These types of occasional and transient pain were still present at the same intensity in the posttrauma period with the exception of abdominal and muscular pain, which decreased.

Posttraumatic headache (PTHA) was assessed with the MIDAS Questionnaire, and 34 (36%) patients reported PTHA, with an average VAS of 60/100 mm lasting for an average of 10 days per month.

As presented in Table 2a, mTBI patients with pain rated the pain intensity at 51.3/100 (±24.9) mm (mean ± SD) on the VAS compared to 23.4/100 (SD ± 31.6) for mTBI without trauma-related pain and 0.59 (SD ± 1.4) in controls, *P* < 0.0001. The mTBI with pain group (*n* = 65; 69.2%) reported more severe symptoms on the Rivermead Post-Concussion Symptoms Questionnaire (22.0 ± 12.7 vs. 13.2 ± 9.9; *P* = 0.003), lower quality of life on the SF-36 questionnaire (47.1 ± 16.4 vs. 72.1 ± 17.0; *P* < 0.0001), higher bodily pain scores on the SF-36 (34.4 ± 17.8 vs. 77.0 ± 19.7; *P* < 0.0001), higher overall and component scores on the Pain Catastrophizing Scale (15.2 ± 12.3 vs. 9.9 ± 7.5; *P* = 0.05), more symptoms of depression on the Beck Depression Inventory (BDI; 12.9 ± 8.6 vs. 5.5 ± 4.3; *P* < 0.0001), more anxiety symptoms on the Beck Anxiety Inventory (BAI) scores (10.2 ± 9.3 vs. 3.7 ± 3.1; *P* = 0.003) and more posttraumatic stress symptom scores on the Impact of Event Scale–Revised (IES-R; 20.2 ± 16.6 vs. 9.4 ± 7.7; *P* = 0.006). Healthy controls presented much lower scores on all administered questionnaires than mTBI patients in general and mTBI patients with pain in particular. However, the difference between controls and mTBI patientswithout pain was not statistically significant for bodily pain SF-36, pain catastrophizing, depression, and anxiety. mTBI patients without pain were significantly more likely to be male (χ^2^ = 4.6; *d* = 1; *P* = 0.05) and returned to work faster than mTBI patients with pain (χ^2^ = 6.6; *d* = 1; *P* = 0.01; OR = 3.9; 95% CI, 1.32, 11.63).

**Table 2.** Description of post-concussion symptoms.

		mTBI with pain (*n* = 65)	mTBI without pain (*n* = 29)	Controls (*n* = 22)	*P* value^b^
(a) Patients’ self-report questionnaires in the acute phase^a^					
Demography	Gender (F/M)	(22/43)	(5/24)	(13/9)	
	Age^c^	38.04 (12.41)	38.88 (12.43)	31.9 (11.91)	**0.055**
Pain-related questionnaires	Pain VAS (100 mm)	51.30 (24.87)	23.37 (31.63)	0.59 (1.40)	**<0.0001**
	Pain Catastrophizing Scale^d^	15.21 (12.26)	9.88 (7.46)	7.76 (5.78)	**0.01**
	Bodily pain SF-36^d^	34.41 (17.76)	77.04 (19.68)	79.44 (23.06)	**<0.0001**
Post-concussion symptoms, psychological questionnaires, quality of life	Rivermead	21.98 (12.74)	13.17 (9.88)	—	**0.003**
	SF-36	47.05 (16.42)	72.09 (16.96)	84.94 (12.15)	**<0.0001**
	BDI-II^d^	12.91 (8.55)	5.48 (4.33)	2.0 (4.29)	**<0.0001**
	BAI^d^	10.17 (9.28)	3.67 (3.14)	2.18 (5.27)	**0.003**
	IES-R	20.16 (16.58)	9.35 (7.71)	—	**0.006**
Sleep–wake disturbances	Fatigue scale^d^	3.4(1.3)	2.4(1.2)	2.5(1.2)	**0.001**
	Sleepiness scale^d^	3.6(1.6)	2.5(1.2)	2.1(1.2)	**<0.0001**
	Mean reaction time^d^	282.2(65.7)	241.0(18.2)	244.6(36.4)	**<0.0001**

		mTBI with chronic pain (*n* = 18)	mTBI without chronic pain (*n* = 18)	*P* value
(b) Patients’ self-report questionnaires in the chronic phase (*n* = 36)^e^				
Pain-related questionnaires	Pain VAS (100 mm)	36.18 (19.49)	6.25 (15.44)	**<0.0001**
	Pain Catastrophizing Scale	9.86 (9.35)	4.88 (5.01)	ns
	Bodily pain SF-36	52.93 (25.37)	77.88 (20.42)	**0.01**
Post-concussion symptoms, psychological questionnaires, quality of life	Rivermead	17.86 (11.88)	7.13 (6.80)	**0.007**
	SF-36	67.00 (19.55)	88.18 (6.94)	**0.005**
	BDI-II	10.22 (10.46)	2.55 (3.36)	**0.02**
	BAI	8.67 (12.24)	2.18 (3.60)	**0.04**
	IES-R	7.81 (15.28)	5.92 (6.92)	ns
Sleep–wake disturbances	Fatigue scale	2.0(1.3)	2.65(1.4)	ns
	Sleepiness scale	1.85(1.0)	2.6(1.4)	ns
	Mean reaction time	318.7(137.6)	24.9(19)	ns

^a^Analysis of variance between mTBI patients with pain and mTBI patients without pain and healthy controls in the acute posttrauma phase (at 6 weeks). Results are shown as mean (SD). Lower scores on the SF-36 and bodily pain SF-36 indicate better quality of life. Rivermead and IES-R were not administered to healthy controls.

^b^The *P* value refers to the analysis of variance between groups.

^c^No statistically significant difference in age among mTBIs but only between controls and mTBI.

^d^No statistically significant difference between mTBI without pain and healthy controls.

^e^A Student’s *t* test comparison between mTBI patients with persistent and newly developed pain vs. mTBI patients without pain at one year posttrauma. Lower scores on the SF-36 and bodily pain SF-36 indicate better quality of life.

mTBI = mild traumatic brain injury; VAS = Visual Analogue Scale; SF-36 = Short-Form 36; BDI = Beck Depression Inventory–II; BAI = Beck Anxiety Inventory; IES-R = Impact of Event Scale–Revised.

### Sleepiness, fatigue, and mean reaction time

mTBI patients with pain present more sleepiness (pain: 3.6/7 ± 1.6; no pain: 2.5/7 ± 1.2; controls: 2.1/7 ± 1.2; *P* ≤ 0.0001), fatigue (pain: 3.4/7 ± 1.3; no pain: 2.4/7 ± 1.2; controls: 2.5/7 ± 1.2; *P* = 0.001) and have a longer reaction time on a PVT task than mTBI patients without pain and healthy controls together (pain: 282.2 ± 65.7 ms; no pain: 241.0 ± 18.2 ms; controls: 244.6 ± 36.4 ms; *P* < 0.0001). There was no statistically significant difference between mTBI patients without pain and controls (Table 2a).

### Genetic association

A total of 31 SNPs within 12 genes were genotyped using Sequenom IPLEX Gold Technology with an average call rate of 99% and all SNPs passed the Hardy-Weinberg equilibrium test.

All available phenotypes cited above were tested for association with the 31 SNPs. The phenotypes included three main categories: pain-related post-concussion symptoms, psychological and quality of life–related phenotypes, and sleep–wake disturbance–related phenotypes. Full data are presented in Supplemental Table S1. From the entire list of 12 genes, only *BDNF* and *DRD2/ANKK1* SNPs were significantly associated with some phenotypes. Five SNPs from *BDNF* passed the correction for multiple testing for association with Rivermead, SF-36, bodily pain SF-36, IES-R, BAI, and fatigue, whereas rs1800497 in *DRD2/ANKK1* was associated with increased sleepiness. Three SNPs in the *BDNF* gene have minor alleles that confer deleterious effects (rs7124442, rs7127507, and rs11030121), whereas two SNPs, including val66met, were protective (rs6265 and rs11030104).

**Figure 1.**
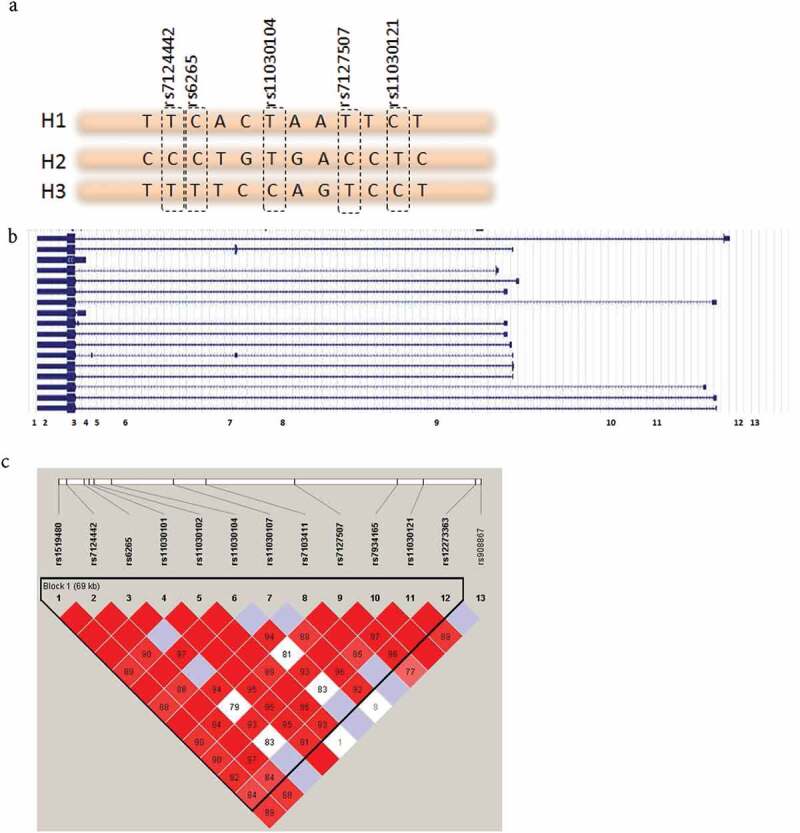
(a) Alleles of SNPs are represented in a short stretch of DNA. Combinations of SNPs used to construct haplotypes are highlighted. (b) BDNF gene structure from refSeq. Full boxes are exons, lines represent introns. Numbers (1–13) represent SNP locations in the gene. (c) LD plot of the SNPs spanning the *BDNF* gene. The numbers correspond to *D*′ values between SNPs. One block of 69 kbp was constructed with Haploview 4.2 using the solid spine method.

Because multiple SNPs within the *BDNF* gene locus were associated with pain phenotypes but only one SNP with only one phenotype was associated with the *DRD2/ANKK1* gene locus, we concentrated on the *BDNF* gene locus. We first tested whether the effect of SNPs was a mere reflection of functional val/met polymorphism or whether additional functional SNPs exist with the *BDNF* gene locus. An LD analysis showed that all SNPs in the *BDNF* gene (except rs908867) span a one-block region of 69 kb (Figure 1).

We constructed haplotypes within this block using the top five most statistically significant SNPs (Supplementary Table S2) with frequency distributions of more than 1% and chose to retain the top three haplotypes because they account for more than 94.5% of our sample. Conditional haplotype testing was applied to the haplotype composed of rs7124442|rs6265|rs11030104|rs7127507|rs11030121 with the following frequencies: H1: TCTTC (46.8%), H2: CCTCT (27%), and H3: TTCTC (20.7%), where haplotypes H2 and H3 have mirror allelic composition. Results show that H1 is not associated with any phenotype. H2 is associated with more pain and more post-concussive symptoms and hence has a deleterious effect, and H3 is associated with less pain, better quality of life, and less fatigue and sleepiness and hence is protective (Table 3). The direction of association continues to be true for haplotypes H2 and H3 even for phenotypes that did not reach significance after correction for multiple comparisons.

**Table 3.** Haplotypes frequency distribution and association with pain status and all phenotypes^a^.

		H1		H2		H3	
		TCTTC		CCTCT		TTCTC	
		0.47		0.27		0.21	
		OR/beta	*P* value^b^	OR/beta	*P* value^b^	OR/beta	*P* value^b^
mTBI with pain vs. mTBI without pain		1.80	0.46	**2.83**	**0.006**	0.55	0.09
Pain-related questionnaires	Pain VAS	−2.05	0.69	6.854	0.25	−4.68	0.53
	Pain Catastrophizing Scale	−1.285	0.42	**5.495**	**0.004**	−3.497	0.08
	Body pain SF-36	0.453	0.90	**−14.6**	**0.007**	**17.85**	**0.001**
Post-concussion symptoms, psychological questionnaires, quality of life	Rivermead	−2.897	0.29	**6.453**	**0.008**	−4.924	0.056
	SF-36	2.005	0.56	**−11.71**	**0.004**	**13.25**	**0.002**
	BDI-II	−1.019	0.44	3.287	0.04	−3.708	0.03
	BAI	−2.653	0.05	**4.255**	**0.006**	−2.814	0.08
	IES-R	−4.296	0.10	**7.121**	**0.02**	−4.873	0.15
Sleep–wake disturbances	Fatigue scale	−0.1307	0.59	0.553	0.04	**−0.683**	**0.01**
	Sleepiness scale	0.2238	0.24	0.155	0.51	**−0.675**	**0.004**
	Mean reaction time	−15.95	0.15	28.46	0.03	−2.621	0.85
			No effect		**Deleterious**		**Protective**

^a^Statistically significant results are shown in bold. Haplotypes are constructed using rs7124442|rs6265|rs11030104|rs7127507|rs11030121. Frequencies of haplotype are shown under each haplotype. Haplotype H2 is deleterious as carriers report more pain, post-concussion symptoms, poor quality of life, posttraumatic stress, anxiety, and pain catastrophizing. On the other hand, H3 is protective with less pain, better quality of life, and less fatigue and sleepiness. Shown are *P* values of association between the haplotypes and each phenotype. Significant *P* value set at ≤0.02. OR = odds ratio; mTBI = mild traumatic brain injury; VAS = Visual Analogue Scale; SF-36 = Short-Form 36; BDI = Beck Depression Inventory–II; BAI = Beck Anxiety Inventory; IES-R = Impact of Event Scale–Revised.

### Expression analysis

A GTex database search for brain, nerve, and blood eQTL as well as DRG eQTL data showed that the top five SNPs included in haplotypes H1, H2, and H3 modulate mRNA expression of *BDNF* and its antisense *BDNF-AS*. We found none of the eQTLs in the blood; however, rs11030121, rs7124442, and rs7127507, which are markers of haplotype H2, are associated with a decreased expression of *BDNF-AS* as well as an increase in the expression of *BDNF* in the DRG. On the other hand, markers of haplotypes H3, rs6265, and rs11030104 are associated with decreased *BDNF-AS* in the cortex and nerves as well as a decrease in *BDNF* in nerves (Table 4a).

**Table 4.** Expression data for top five SNPs in BDNF in public databases and mTBI patients^a^.

(a) eQTL data retrieved from DRG and GTeX databases									
			DRG		Cortex		Nerve		
Markers	SNP	MA	Beta	*P* value	Beta	*P* value	Beta	*P* value	Gene
H2	rs7124442	C	**−0.038**	**0.002**	−0.05	0.54	0.066	0.14	*BDNF-AS*
	rs7127507	C	**−0.038**	**0.003**	0.027	0.74	−0.077	0.09	*BDNF-AS*
	rs11030121	T	**−0.040**	**0.002**	0.062	0.46	−0.068	0.13	*BDNF-AS*
	rs7124442	C	**0.043**	**0.017**	0.13	0.14	−0.043	0.55	*BDNF*
	rs7127507	C	**0.045**	**0.013**	−0.15	0.10	0.021	0.77	*BDNF*
	rs11030121	T	**0.047**	**0.010**	−0.098	0.27	0.034	0.65	*BDNF*
H3	rs6265	T	−0.010	0.53	**−0.331**	**0.003**	**−0.179**	**0.001**	*BDNF-AS*
	rs11030104	C	−0.004	0.77	**−0.335**	**0.002**	**−0.189**	**3.6E-04**	*BDNF-AS*
	rs6265	T	−0.008	0.71	0.026	0.83	**−0.409**	**5.7E-06**	*BDNF*
	rs11030104	C	0.0003	0.98	0.065	0.57	**−0.415**	**1.3E-06**	*BDNF*

(b) BDNF-AS and BDNF expression from immortalized lymphoblastoid cell lines of patients						
	H1		H2		H3	
	TCTTC		CCTCT		TTCTC	
Frequency of haplotypes	**0.47**		**0.27**		**0.21**	
	Beta	*P* value	Beta	*P* value	Beta	*P* value
Delta Ct *BDNF-AS*	−0.62	0.11	−0.19	0.68	**1.107**	**0.02**
Delta Ct *BDNF*	−0.12	0.58	0.234	0.35	0.06	0.83

^a^Statistically significant eQTLs are shown in bold. Three SNPs, markers of H2, control the expression of *BDNF* and *BDNF-AS* in opposite directions in DRG. The two SNP markers of H3 control the expression of *BDNF-AS* in the brain and nerves and *BDNF* only in nerves. Haplotypes are constructed using rs7124442|rs6265|rs11030104|rs7127507|rs11030121. Higher delta Ct represents lower mRNA expression.

SNP = single nucleotide polymorphism; BDNF = brain-derived neurotrophic factor; mTBI = mild traumatic brain injury; eQTL = expression quantitative trait loci; DRG = dorsal root ganglia; Minor allele (MA) = BDNF-AS = anti-sense of *BDNF*.

**Figure 2.**
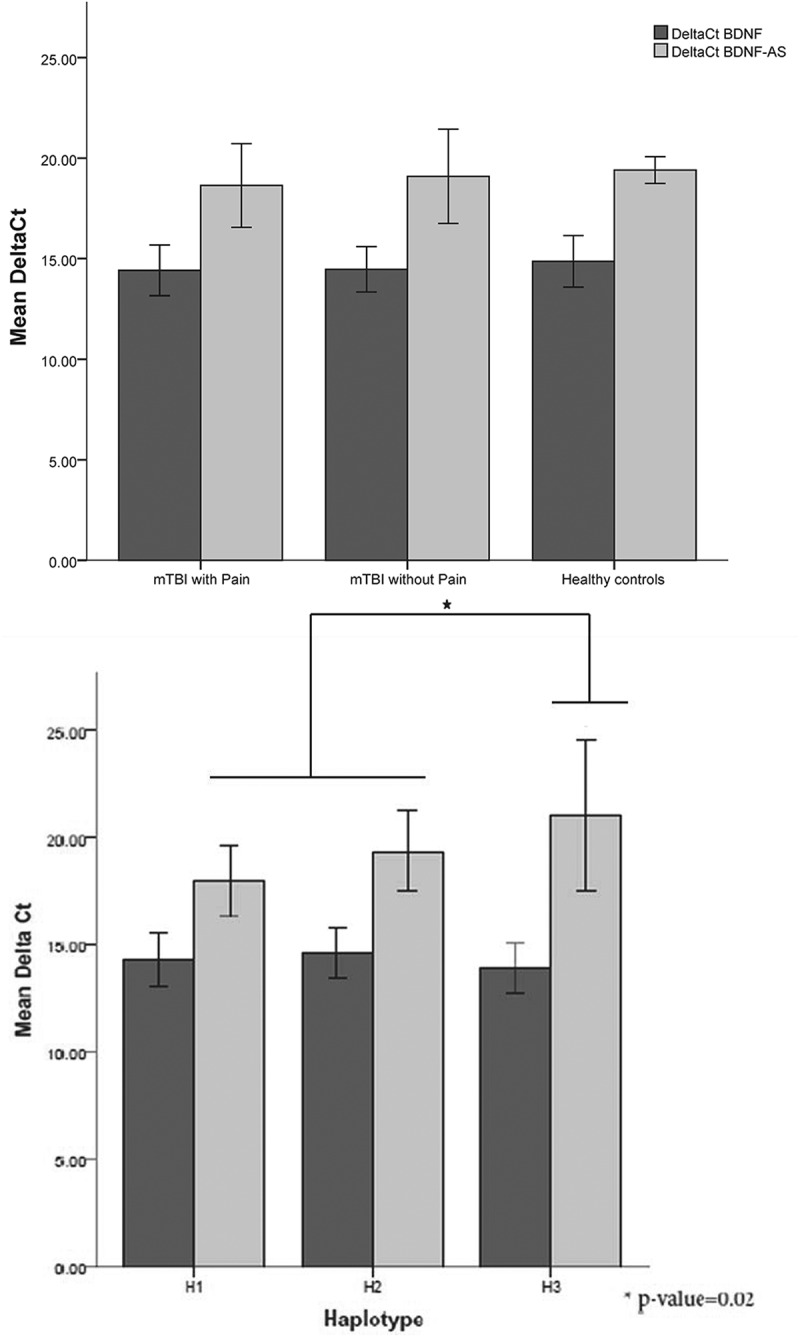
(a) qPCR results of *BDNF* and *BDNF-AS* mRNA expression relative to GAPDH (delta Ct) comparison between mTBI patients with pain and without pain and healthy controls. (b) qPCR results of *BDNF* and *BDNF-AS* mRNA expression relative to GAPDH (delta Ct) comparison between the three haplotypes H1 (*n* = 44), H2 (*n* = 25), and H3 (*n* = 20). *Corresponds to the significant group difference between *BDNF-AS* level of H3 compared to *BDNF-AS* levels of H1 and H2. Haplotypes are composed from rs7124442|rs6265|rs11030104|rs7127507|rs11030121.

To assess expression of *BDNF* as well as *BDNF-AS* for each haplotype, immortalized lymboblastoid cell lines isolated from mTBI patients with pain and without pain and healthy controls were used. *BDNF* and *BDNF-AS* mRNA expression showed no statistically significant difference between mTBI patients with pain or without pain and control subjects (Figure 2a). Carriers of haplotype H3 have less *BDNF-AS* than H1 and H2, without any difference for *BDNF* (Table 4b, Figure 2b).

### Prospective pilot study, one-year follow-up

In this pilot study, 36 mTBI patients (38% of the initial sample; 23 males, 13 females; mean age 45.1 ± 11.6 years) agreed to participate in the one-year follow-up (mean = 429.9 ± 59.4 days posttrauma). After one year, ten remained pain free, eight had no more pain, three had developed new onset pain (head and neck pain), and 15 still had (persistent) pain. Overall, 18 out of the 36 of mTBI patients reported chronic pain, with a mean pain VAS score of 36.2/100 mm (SD ± 19.5), compared to mTBI patients without pain, mean VAS score of 6.3/100 (SD ± 15.4); *P* < 0.0001.

At the follow-up, mTBI patients with chronic pain (*n* = 18) reported more severe symptoms on the Rivermead Post-Concussion Symptoms Questionnaire compared to mTBI patients without chronic pain (17.9 ± 11.9 vs. 7.1 ± 6.8; *P* = 0.007), lower quality of life on the SF-36 questionnaire (67.0 ± 19.6 vs. 88.2 ± 6.9; *P* = 0.005), more bodily pain on the SF-36 (52.9 ± 25.4 vs. 77.9 ± 20.4; *P* = 0.01), higher depression scores on the BDI (10.2 ± 10.5 vs. 2.6 ± 3.4; *P* = 0.02), and higher anxiety scores on the BAI (8.7 ± 12.2 vs. 2.2 ± 3.6; *P* = 0.04). The Pain Catastrophizing Scale score and the Impact of Event Scale showed no statistical difference between the two groups at follow-up, even if they were higher in the pain group. No differences were found on sleepiness, fatigue, and mean reaction time on the PVT for both groups (Table 2b).

**Figure 3.**
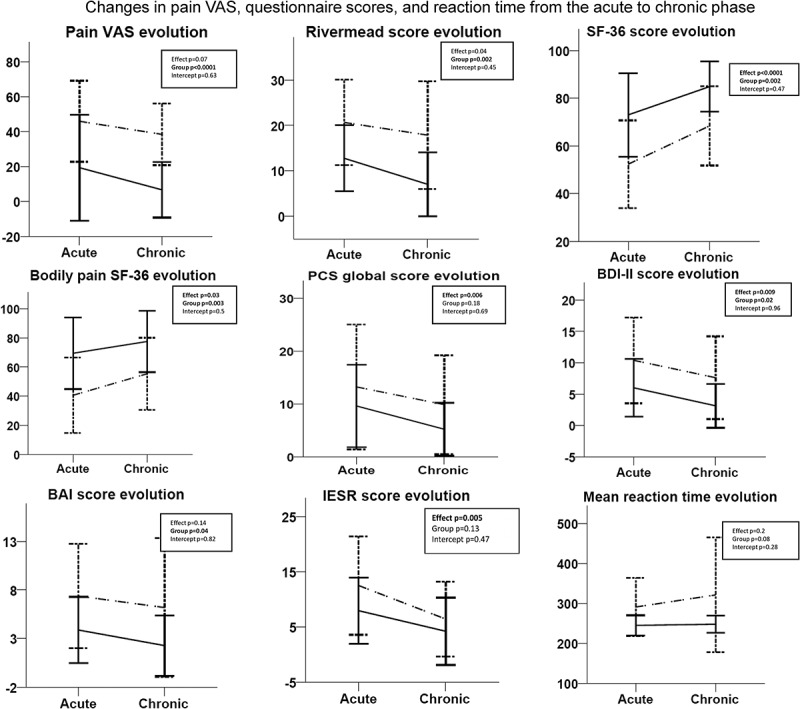
Comparison between acute and chronic questionnaire (Rivermead, SF-36, Pain Catastrophizing Scale, Beck Depression Inventory, Beck Anxiety Inventory, Impact of Event Scale) scores, pain VAS, and mean reaction time on the PVT. A repeated measures ANOVA was used. Dotted lines represent mTBI patients with pain (*n* = 18); full lines represent mTBI patients without pain (*n* = 18).

Changes in symptoms in mTBI patients with chronic pain at one year posttrauma are illustrated in Figure 3. Overall, Rivermead, Pain Catastrophizing Scale, and BDI scores decreased for mTBI patients with and without pain. SF-36 and bodily pain SF-36 scores increased, reflecting improved quality of life. These results indicate that mTBI patients with persistent or newly developed pain show fewer post-concussion symptoms, less depression and anxiety, less pain-related catastrophizing, and better quality of life than in the acute posttrauma phase. No differences were found on mean reaction time on the PVT between the acute and chronic phase for both groups.

### Genetic factors, one-year follow-up

In this pilot analysis, *BDNF* haplotype frequencies were assessed in persistent or newly developed pain and in mTBI patients with resolved or no pain.

mTBI patients with chronic pain were more likely to carry haplotype H2 (χ^2^ = 4.8; *d* = 1; OR = 4.47; 95% CI, 1.1, 29.8; *P* = 0.001), whereas carriers of haplotype H3 are protected against development of chronic pain (χ^2^ = 10.5; *d* = 1; OR = 0.10; 95% CI, 0.09, 0.81; *P* = 0.03). The frequencies of H1, H2, and H3 respectively differed in mTBI with persistent or newly developed pain versus mTBI with no pain (H1: 49.8% vs. 46.7%, *P* = 0.8; H2: 37.6% vs. 13.3%; *P* = 0.03; H3: 3.8% vs. 36.7%, *P* = 0.001). Due to the low sample size (*n* = 18 per group), these results are preliminary.

## Discussion

### Psychological and pain variables in the acute and chronic phases

In the acute phase following mTBI, around 70% of patients report pain, mainly in the form of posttraumatic headaches. Psychological factors such as depression and anxiety were more prevalent in mTBI patients with pain than in those without pain. Post-concussion symptoms, measured using the Rivermead Post-Concussion Symptoms questionnaire, were also more severe in the presence of pain. Because of pain, quality of life and return to work and usual activities were compromised in the acute phase following mTBI. These results are in line with previous findings that mTBI patients report more depression, anxiety, pain catastrophizing, and sleep disturbances than healthy individuals and that these symptoms were exacerbated in the presence of pain.^6^ It is also important to note that mTBI patients without pain reported depression and anxiety symptoms with the same intensity as healthy controls without a history of trauma, as well as some pain on the VAS scale. These results show that even if patients are not classified in the pain group by research criteria, they still suffer from post-concussion symptoms that will resolve eventually, as seen in the prospective pilot arm of this study. On the other hand, the relationship between sleep and pain in this cohort fits with the generally accepted dogma that mTBI patients with pain present symptoms of sleepiness and fatigue as well as decreased vigilance, whereas the absence of pain did not affect these symptoms. This observation underlines the importance of pain in posttraumatic sleep–wake disturbances.

The most common type of pain reported in both acute and chronic mTBI phases was PTHA. A similar finding was reported for moderate to severe long-term survivor TBI patients.^60^ Chronic pain may be part of a larger family of post-concussion symptoms rather than being isolated and should be treated as part of a group of symptoms.

### BDNF and TBI outcome

Many associations between *BDNF* polymorphisms, mainly val66met, and outcomes following TBI were investigated in previous studies.^24,52,61,62^ Variations in *BDNF* were shown to explain general intelligence on cognitive and executive tasks many years after combat-related penetrating TBI.^24,62^ Another study found an association between memory and processing speed following mTBI and *BDNF*.^52^ Other report showed that the val/val genotype was associated with experience dependent plasticity in the motor cortex.^32^ Finally, recently, the met genotype was associated with better olfactory functions following concussion.^63^ This study broadens the contribution of genetic variability within the *BDNF* gene locus to pain and post-concussion symptoms by finding that val/met polymorphism is not the only important variant in outcome following mTBI but that there are other functional polymorphisms in the same haploblock. In fact, the haplotype analysis presented in this article shows that two of three haplotypes are important for pain post-concussion in both the acute and chronic phases.

The structure of haplotypes H2 and H3 is interesting and very informative; for instance, each haplotype’s markers are composed of tag SNPs that show deleterious or protective effects. Markers of haplotype H2 are associated with chronic pain, sleepiness, fatigue, poor quality of life, and post-concussive phenotypes, whereas haplotype H3 is protective against post-concussive symptoms and pain.

In the present study, we show that haplotype H3, which has methionine as a marker, decreases the expression of anti-sense BDNF (*BDNF-AS*), which is known to inhibit BDNF mRNA expression, possibly through RNA duplexes.^27,29^ We could then speculate that the H3 haplotype, which includes the met allele, is protective because it disinhibits BDNF expression and favors BDNF release. In lymphoblastoid cell lines, however, we were not able to demonstrate an increase in BDNF, due to the low expression of BDNF in these cells as well as the low sample size. When our results are compared to public eQTL databases, the same pattern of expression is observed. Therefore, genetic susceptibility in factors regulating brain plasticity may play an important role in neuronal recovery following mTBI. In this article, we introduce the concept that there is a multilocus effect of *BDNF* haplotypes affecting post-concussion symptoms. This effect may be driven by the antisense, leading to beneficial BDNF presence.

It is well known that *BDNF* and *BDNF-AS* are expressed in a tissue-specific manner and in a distinct, although partially overlapping, manner. Both are highly expressed in the brain as well as in neuronal tissues but to a lesser extent in blood.^27^ This difference in tissue expression could explain the low levels that we were able to detect in blood that is nevertheless consistent with neuronal tissues.

### Factors predisposing to chronicity of pain

At one year posttrauma, almost 38% of the original participants agreed to participate in the follow-up, and 19% of these still suffer from pain. The results show that at one year posttrauma, mTBI patients with persistent or newly developed chronic pain show fewer symptoms than in the acute phase but also report lower quality of life, persistent post-concussion symptoms, and more severe depression and anxiety compared to patients without pain. More interesting, three patients (8%) developed pain that was absent in the acute posttrauma phase. A possible explanation is that pain in the acute phase was well controlled and therefore not reflected in the questionnaires or medical reports.

The most significant finding is that haplotype H2 is a predictor of persistent or newly developed chronic pain in mTBI patients and carriers of haplotype H3 are protected from the development of chronicity. To our knowledge, this is the first study to demonstrate the role of multiple loci in *BDNF* for chronic post-mTBI pain; however, due to low sample size, these are only preliminary findings.

### Study limitations

Our study has certain limitations that need to be addressed. First, despite considerable efforts, the one-year follow-up rate of recruited patients was relatively low at 38%. The difficulties in recruitment in TBI studies are a subject addressed in a growing body of literature with proposed solutions to be implemented in the future,^64–66^ because this low recruitment rate can introduce a bias in the results and weaken the development of knowledge within the field. Second, we used psychomotor vigilance testing to assess mean reaction time. Although it has been validated in sleep deprivation studies, we were unable to find a validation study in a cohort of mTBI patients, which limited our ability to reference its use in this population. Third, *BDNF* expression was measured in lymphoblastoid cell line because they were the only resources available to us. Though endogenous expression of BDNF was confirmed in these cells, neuronal or glial cell lines would have been more appropriate.^67^ Last, the sample size of this study is too small to draw definitive conclusions on the association between *BDNF* and pain related outcomes in TBI; however, the results presented should be taken into consideration for future trials with larger cohorts.

### Conclusions

This study shows that *BDNF-AS* is key for the protective effect of *BDNF* haplotypes through a multilocus effect against post-concussion symptoms and pain in the acute phase and, most important, for the development of chronic pain following mTBI. Future studies should focus on mechanisms of inhibition of *BDNF-AS* as a potential therapeutic target. Increasing evidence thus points to an immediate *BDNF* response in the central nervous system following trauma. The identification of at-risk patients as well as the consequences of better control of post-concussion symptoms in the acute posttrauma phase should be considered when assessing mTBI.

## Supplementary Material

UCJP_A_1362942_supplemental_material.docx

## Supplemental data

Supplemental data for this article can be access on the publisher’s website.

## References

[1] Cassidy JD, Carroll LJ, Peloso PM, Borg J, Van Holst H, Holm L, Kraus J, Coronado VG. Incidence, risk factors and prevention of mild traumatic brain injury: results of the WHO collaborating centre task force on mild traumatic brain injury. J Rehabil Med. 2004;36(Suppl. 43):28–60. doi:10.1080/16501960410023732. 15083870

[2] Feinstein A, Rapoport M. Mild traumatic brain injury: the silent epidemic. Can J Public Health. 2000;91(5):325–326. 11089281 10.1007/BF03404799 PMC6979787

[3] Zumstein MA, Moser M, Mottini M, Ott SR, Sadowski-Cron C, Radanov BP, Zimmermann H, Exadaktylos A. Long-term outcome in patients with mild traumatic brain injury: a prospective observational study. J Trauma. 2011;71(1):120–127. doi:10.1097/TA.0b013e3181f2d670. 21045743

[4] Risdall JE, Menon DK. Traumatic brain injury. Philos Trans R Soc Lond B Biol Sci. 2011;366(1562):241–250. doi:10.1098/rstb.2010.0230. 21149359 PMC3013429

[5] Nampiaparampil DE. Prevalence of chronic pain after traumatic brain injury: a systematic review. JAMA. 2008;300(6):711–719. doi:10.1001/jama.300.6.711. 18698069

[6] Khoury S, Chouchou F, Amzica F, Giguère JF, Denis R, Rouleau GA, Lavigne GJ. Rapid EEG activity during sleep dominates in mild traumatic brain injury patients with acute pain. J Neurotrauma. 2013;30(8):633–641. doi:10.1089/neu.2012.2519. 23510169

[7] Baumann CR. Traumatic brain injury and disturbed sleep and wakefulness. Neuromolecular Med. 2012;14(3):205–212. doi:10.1007/s12017-012-8178-x. 22441999

[8] Lavigne G, Khoury S, Chauny JM, Desautels A. Pain and sleep in post-concussion/mild traumatic brain injury. Pain. 2015;156(Suppl. 1):S75–S85. doi:10.1097/j.pain.0000000000000111. 25789439

[9] Suzuki Y, Khoury S, El-Khatib H, Chauny JM, Paquet J, Giguère JF, Denis R, Gosselin N, Lavigne GJ, Arbour C. Individuals with pain need more sleep in the early stage of mild traumatic brain injury. Sleep Med. 2016;33:36–42. 28449903 10.1016/j.sleep.2016.06.033

[10] Sullivan KA, Edmed SL, Allan AC, Karlsson LJ, Smith SS. Characterizing self-reported sleep disturbances after mild traumatic brain injury. J Neurotrauma. 2014;32(7):474–86. 10.1089/neu.2013.3284 PMC4376482 25275933

[11] Arbour C, Khoury S, Lavigne GJ, Gagnon K, Poirier G, Montplaisir JY, Carrier J, Gosselin N. Are NREM sleep characteristics associated to subjective sleep complaints after mild traumatic brain injury? Sleep Med. 2015;16(4):534–539. doi:10.1016/j.sleep.2014.12.002. 25747335

[12] Radanov BP, Di Stefano G, Schnidrig A, Ballinari P. Role of psychosocial stress in recovery from common whiplash. Lancet. 1991;338(8769):712–715. doi:10.1016/0140-6736(91)91441-V. 1679865

[13] Stulemeijer M, Van Der Werf S, Borm GF, Vos PE. Early prediction of favourable recovery 6 months after mild traumatic brain injury. J Neurol Neurosurg Psychiatry. 2008;79:936–942. doi:10.1136/jnnp.2007.131250. 17951281

[14] Mott TF, McConnon ML, Rieger BP. Subacute to chronic mild traumatic brain injury. Am Fam Physician. 2012;86(11):1045–1051. 23198672

[15] DeKosky ST, Blennow K, Ikonomovic MD, Gandy S. Acute and chronic traumatic encephalopathies: pathogenesis and biomarkers. Nat Rev Neurol. 2013;9(4):192–200. doi:10.1038/nrneurol.2013.36. 23558985 PMC4006940

[16] Otis JD, McGlinchey R, Vasterling JJ, Kerns RD. Complicating factors associated with mild traumatic brain injury: impact on pain and posttraumatic stress disorder treatment. J Clin Psychol Med Settings. 2011;18:145–154. doi:10.1007/s10880-011-9239-2. 21626354

[17] Radresa O, Chauny JM, Lavigne GJ, Piette E, Paquet J, Daoust R. Current views on acute to chronic pain transition in post-traumatic patients: risk factors and potential for pre-emptive treatments. J Trauma Acute Care Surg. 2014;76(4):1142–1150. doi:10.1097/TA.0000000000000188. 24662883

[18] Dardiotis E, Grigoriadis S, Hadjigeorgiou GM. Genetic factors influencing outcome from neurotrauma. Curr Opin Psychiatry. 2012;25(3):231–238. doi:10.1097/YCO.0b013e3283523c0e. 22449762

[19] McAllister TW. Genetic factors modulating outcome after neurotrauma. PM R. 2010;2(Suppl. 12):S241–S252. doi:10.1016/j.pmrj.2010.10.005. 21172686

[20] Feala JD, Abdulhameed MD, Yu C, Dutta B, Yu X, Schmid K, Dave J, Tortella F, Reifman J. Systems biology approaches for discovering biomarkers for traumatic brain injury. J Neurotrauma. 2013;30(13):1101–1116. doi:10.1089/neu.2012.2631. 23510232 PMC3700463

[21] Tierney RT, Mansell JL, Higgins M, McDevitt JK, Toone N, Gaughan JP, Mishra A, Krynetskiy E. Apolipoprotein E genotype and concussion in college athletes. Clin J Sport Med. 2010;20(6):464–468. doi:10.1097/JSM.0b013e3181fc0a81. 21079443

[22] Minambres E, Cemborain A, Sanchez-Velasco P, Gandarillas M, Diaz-Reganon G, Sanchez-Gonzalez U, Leyva-Cobian F. Correlation between transcranial interleukin-6 gradient and outcome in patients with acute brain injury. Crit Care Med. 2003;31(3):933–938. 12627008 10.1097/01.CCM.0000055370.66389.59

[23] Lipsky RH, Sparling MB, Ryan LM, Xu K, Salazar AM, Goldman D, Warden DL. Association of COMT Val158Met genotype with executive functioning following traumatic brain injury. J Neuropsychiatry Clin Neurosci. 2005;17(4):465–471. doi:10.1176/jnp.17.4.465. 16387984

[24] Rostami E, Krueger F, Zoubak S, Dal Monte O, Raymont V, Pardini M, Hodgkinson CA, Goldman D, Risling M, Grafman J. BDNF polymorphism predicts general intelligence after penetrating traumatic brain injury. PLoS One. 2011;6(11):e27389. doi:10.1371/journal.pone.0027389. 22087305 PMC3210804

[25] McAllister TW, Flashman LA, Harker Rhodes C, Tyler AL, Moore JH, Saykin AJ, McDonald BC, Tosteson TD, Tsongalis GJ. Single nucleotide polymorphisms in ANKK1 and dopamine D2 receptor gene affect cognitive outcome shortly after traumatic brain injury: a replication and extension study. Brain Inj. 2008;22(9):705–714. doi:10.1080/02699050802263019. 18698520 PMC3169811

[26] McAllister TW, Rhodes CH, Flashman LA, McDonald BC, Belloni D, Saykin AJ. Effect of the dopamine D2 receptor T allele on response latency after mild traumatic brain injury. Am J Psychiatry. 2005;162(9):1749–1751. doi:10.1176/appi.ajp.162.9.1749. 16135640

[27] Pruunsild P, Kazantseva A, Aid T, Palm K, Timmusk T. Dissecting the human BDNF locus: bidirectional transcription, complex splicing, and multiple promoters. Genomics. 2007;90:397–406. doi:10.1016/j.ygeno.2007.05.004. 17629449 PMC2568880

[28] Egan MF, Kojima M, Callicott JH, Goldberg TE, Kolachana BS, Bertolino A, Zaitsev E, Gold B, Goldman D, Dean M, et al. The BDNF Val66Met polymorphism affects activity-dependent secretion of BDNF and human memory and hippocampal function. Cell. 2003;112:257–269. doi:10.1016/S0092-8674(03)00035-7. 12553913

[29] Modarresi F, Faghihi MA, Lopez-Toledano MA, Fatemi RP, Magistri M, Brothers SP, Van Der Brug MP, Wahlestedt C. Inhibition of natural antisense transcripts in vivo results in gene-specific transcriptional upregulation. Nat Biotechnol. 2012;30(5):453–459. doi:10.1038/nbt.2158. 22446693 PMC4144683

[30] Pezet S, McMahon SB. Neurotrophins: mediators and modulators of pain. Annu Rev Neurosci. 2006;29:507–538. doi:10.1146/annurev.neuro.29.051605.112929. 16776595

[31] Lu B. BDNF and activity-dependent synaptic modulation. Learn Mem. 2003;10(2):86–98. doi:10.1101/lm.54603. 12663747 PMC5479144

[32] Kleim JA, Chan S, Pringle E, Schallert K, Procaccio V, Jimenez R, Cramer SC. BDNF val66met polymorphism is associated with modified experience-dependent plasticity in human motor cortex. Nat Neurosci. 2006;9(6):735–737. doi:10.1038/nn1699. 16680163

[33] Poo MM. Neurotrophins as synaptic modulators. Nat Rev Neurosci. 2001;2:24–32. doi:10.1038/35049004. 11253356

[34] Khalin I, Alyautdin R, Wong TW, Gnanou J, Kocherga G, Kreuter J. Brain-derived neurotrophic factor delivered to the brain using poly (lactide-co-glycolide) nanoparticles improves neurological and cognitive outcome in mice with traumatic brain injury. Drug Deliv. 2016;16:1–9. 10.1080/10717544.2016.1199609 27278330

[35] Carroll LJ, Cassidy JD, Holm L, Kraus J, Coronado VG. Methodological issues and research recommendations for mild traumatic brain injury: the WHO collaborating centre task force on mild traumatic brain injury. J Rehabil Med. 2004;36(Suppl. 43):113–125. doi:10.1080/16501960410023877. 15083875

[36] Headache Classification Subcommittee of the International Headache SocietyThe international classification of headache disorders, 2nd edition. Cephalalgia Int J Headache. 2004;24(Suppl. 1):58–64. 10.1111/j.1468-2982.2003.00824.x 14979299

[37] Sullivan MJL, Bishop S, Pivik J. The Pain Catastrophizing Scale: development and validation. Psychol Assess. 1995;7:524–532. doi:10.1037/1040-3590.7.4.524.

[38] Stewart WF, Lipton RB, Whyte J, Dowson A, Kolodner K, Liberman JN, Sawyer J. An international study to assess reliability of the Migraine Disability Assessment (MIDAS) score. Neurology. 1999;53(5):988–994. doi:10.1212/WNL.53.5.988. 10496257

[39] King NS, Crawford S, Wenden FJ, Moss NE, Wade DT. The Rivermead Post Concussion Symptoms Questionnaire: a measure of symptoms commonly experienced after head injury and its reliability. J Neurol. 1995;242:587–92. doi:10.1007/BF00868811. 8551320

[40] Ware JE, Snow KK, Kosinski M, Gandek B. The SF-36 Health Survey. Manual and interpretation guide. Boston (MA): New England Medical Center; 1993.

[41] Beck AT, Ward CH, Mendelson M, Mock J, Erbaugh J. An inventory for measuring depression. Arch Gen Psychiatry. 1961;4:561–571. doi:10.1001/archpsyc.1961.01710120031004. 13688369

[42] Beck AT, Epstein N, Brown G, Steer RA. An inventory for measuring clinical anxiety: psychometric properties. J Consult Clin Psychol. 1998;56:893–897. doi:10.1037/0022-006X.56.6.893. 3204199

[43] Weiss DS, Marmar CR. The Impact of Event Scale–Revised. In: Wilson JP, Keane TM, editors. Assessing psychological trauma and PTSD. New York (NY): Guilford Press; 1997. p. 399–411.

[44] Lim J, Dinges DF. Sleep deprivation and vigilant attention. Ann NY Acad Sci. 2008;1129:305–322. doi:10.1196/annals.1417.002. 18591490

[45] Herscovitch J, Broughton R. Sensitivity of the Stanford Sleepiness Scale to the effects of cumulative partial sleep deprivation and recovery oversleeping. Sleep. 1981;4(1):83–91. doi:10.1093/sleep/4.1.83. 7232973

[46] Ariza M, Pueyo R, Matarin M, Junque C, Mataro M, Clemente I, Moral P, Poca MA, Garnacho A, Sahuquillo J. Influence of APOE polymorphism on cognitive and behavioural outcome in moderate and severe traumatic brain injury. J Neurol Neurosurg Psychiatry. 2006;77:1191–1193. doi:10.1136/jnnp.2005.085167. 16614010 PMC2077553

[47] Terrell TR, Bostick RM, Abramson R, Xie D, Barfield W, Cantu R, Stanek M, Ewing T. APOE, APOE promoter, and Tau genotypes and risk for concussion in college athletes. Clin J Sport Med. 2008;18(1):10–17. doi:10.1097/JSM.0b013e31815c1d4c. 18185033

[48] Sarnaik AA, Conley YP, Okonkwo DO, Barr TL, Fink EL, Szabo C, Kochanek PM, Clark RS. Influence of PARP-1 polymorphisms in patients after traumatic brain injury. J Neurotrauma. 2010;27(3):465–471. doi:10.1089/neu.2009.1171. 19925161 PMC2867630

[49] Tanriverdi T, Uzan M, Sanus GZ, Baykara O, Is M, Ozkara C, Buyra N. Lack of association between the IL1A (−889) polymorphism and outcome after head injury. Surg Neurol. 2006;65:7–10. doi:10.1016/j.surneu.2005.05.024. 16378839

[50] Uzan M, Tanriverdi T, Baykara O, Kafadar A, Sanus GZ, Tureci E, Ozkara C, Uysal O, Buyra N. Association between interleukin-1 beta (IL-1b) gene polymorphism and outcome after head injury: an early report. Acta Neurochir (Wien). 2005;147:715–720. doi:10.1007/s00701-005-0529-z. 15891809

[51] Martinez-Lucas P, Moreno-Cuesta J, Garcia-Olmo DC, Escribano-Martinez J, Cuartero Del Pozo A, Lizan-Garcia M, Garci-Olmo D. Relationship between the Arg72Pro polymorphism of p53 and outcome for patients with traumatic brain injury. Intensive Care Med. 2005;31:1168–1173. doi:10.1007/s00134-005-2715-0. 16007417

[52] McAllister TW, Tyler AL, Flashman LA, Rhodes CH, McDonald BC, Saykin AJ, Tosteson TD, Tsongalis GJ, Moore JH. Polymorphisms in the brain-derived neurotrophic factor gene influence memory and processing speed one month after brain injury. J Neurotrauma. 2012;29(6):1111–1118. doi:10.1089/neu.2011.1930. 22188054 PMC3325555

[53] McDevitt JK, Tierney RT, Mansell JL, Driban JB, Higgins M, Toone N, Mishra A, Krynetskiy E. Neuronal structure protein polymorphism and concussion in college athletes. Brain Inj. 2011;25(11):1108–1113. 21902461 10.3109/02699052.2011.607790

[54] Dellavalle B, Hempel C, Kurtzhals JAL, Penkowa M. In vivo expression of neuroglobin in reactive astrocytes during neuropathology in murine models of traumatic brain injury, cerebral malaria, and autoimmune encephalitis. Glia. 2010;58:1220–1227. 20544857 10.1002/glia.21002

[55] Consortium G. The Genotype–Tissue Expression (GTEx) pilot analysis: multitissue gene regulation in humans. Science. 2015;348(6235):648–660. doi:10.1126/science.1262110. 25954001 PMC4547484

[56] Parisien M, Khoury S, Chabot-Dore AJ, Sotocinal SG, Slade GD, Smith SB, Fillingim RB, Ohrbach R, Greenspan JD, Maixner W, et al. Effect of human genetic variability on gene expression in dorsal root ganglia and association with pain phenotypes. Cell Rep. 2017;19(9):1940–1952. doi:10.1016/j.celrep.2017.05.018. 28564610 PMC5524461

[57] Purcell S, Neale B, Todd-Brown K, Thomas L, Ferreira MAR, Bender D, Maller J, Sklar P, De Bakker PIW, Daly MJ, et al. PLINK: a tool set for whole-genome association and population-based linkage analyses. Am J Hum Genet. 2007;81(3):559–575. doi:10.1086/519795. 17701901 PMC1950838

[58] Nyholt DR. A simple correction for multiple testing for SNPs in linkage disequilibrium with each other. Am J Hum Genet. 2004;74(4):765–769. doi:10.1086/383251. 14997420 PMC1181954

[59] Zaykin DV, Westfall PH, Young SS, Karnoub MC, Wagner MJ, Ehm MG. Testing association of statistically inferred haplotypes with discrete and continuous traits in samples of unrelated individuals. Hum Hered. 2002;53:79–91. doi:10.1159/000057986. 12037407

[60] Brown S, Hawker G, Beaton D, Colantonio A. Long-term musculoskeletal complaints after traumatic brain injury. Brain Inj. 2011;25(5):453–461. doi:10.3109/02699052.2011.556581. 21401368

[61] Bagnato S, Minafra L, Bravata V, Boccagni C, Sant’angelo A, Castiglione A, Andriolo M, Lucca LF, De Tanti A, Pistarini C, et al. Brain-derived neurotrophic factor (Val66Met) polymorphism does not influence recovery from a post-traumatic vegetative state: a blinded retrospective multi-centric study. J Neurotrauma. 2012;29(11):2050–2059. doi:10.1089/neu.2011.2184. 22708958

[62] Krueger F, Pardini M, Huey ED, Raymont V, Solomon J, Lipsky RH, Hodgkinson CA, Goldman D, Grafman J. The role of the Met66 brain-derived neurotrophic factor allele in the recovery of executive functioning after combat-related traumatic brain injury. J Neurosci. 2011;31(2):598–606. doi:10.1523/JNEUROSCI.1399-10.2011. 21228168 PMC3195417

[63] Larson-Dupuis C, Chamard E, Falardeau V, Frasnelli J, Beaulieu C, Poirier J, Carrier J, Lassonde M, Théoret H, Bacon BA, et al. Impact of BDNF Val66Met polymorphism on olfactory functions of female concussed athletes. Brain Inj. 2015;29(7–8):963–970. doi:10.3109/02699052.2015.1016452. 25950261

[64] Li LM, Menon DK, Janowitz T. Cross-sectional analysis of data from the U.S. clinical trials database reveals poor translational clinical trial effort for traumatic brain injury, compared with stroke. PLoS One. 2014;9(1):e84336. doi:10.1371/journal.pone.0084336. 24416218 PMC3885561

[65] Joshi S, Dunbar K, Taylor P, Sullivan KL, Afzal MM, Song C, Purohit M, Roy MJ. Streamlining participant recruitment for TBI and PTSD research studies. Mil Med. 2017;182(S1):124–127. doi:10.7205/MILMED-D-16-00282. 28291463

[66] Menon DK. Unique challenges in clinical trials in traumatic brain injury. Crit Care Med. 2009;37(Suppl.):S129–S135. doi:10.1097/CCM.0b013e3181921225. 19104212

[67] Fauchais AL, Lalloué F, Lise MC, Boumediene A, Preud’homme JL, Vidal E, Jauberteau MO. Role of endogenous brain-derived neurotrophic factor and sortilin in B cell survival. J Immunol. 2008;181(5):3027–3038. doi:10.4049/jimmunol.181.5.3027. 18713973

