# Supplementary material for: Post-concussion symptoms and chronic pain after mild traumatic brain injury are modulated by multiple locus effect in the BDNF gene through the expression of antisense: A pilot prospective control study
Source: Can J Pain. 2017 Sep 13;1(1):112–26. doi: 10.1080/24740527.2017.1362942 (PMC8730664; doi:10.1080/24740527.2017.1362942)
Supplement: UCJP_A_1362942_supplemental_material.docx [file UCJP_A_1362942_SM2003.docx]

**Supplementary table 1- Association of outcomes in acute mTBI with SNPs**

Results highlighted in bold are statistically significant after correction for multiple testing. SNP: Single nucleotide polymorphism; MAF: Minor allelic frequency; VAS: Visual Analogue Scale; IES-R: Impact of event scale – Revised; PCS: Pain Catastrophizing scale; BDI: Beck Depression Inventory-II. BAI: Beck Anxiety Inventory. Minor alleles are shown between parentheses.

**Supplementary table 2- Haplotype frequencies of the 69 kb block using the five top significant SNPs**

| Block | Haplotypes | Frequencies |
| --- | --- | --- |
| **H1** | **TCTTC** | **0.468** |
| **H2** | **CCTCT** | **0.270** |
| **H3** | **TTCTC** | **0.207** |
| H4 | TCTTT | 0.015 |
| H5 | TTCCT | 0.013 |
| H6 | CCTTT | 0.010 |

*rs7124442|rs6265|rs11030104|rs7127507|rs11030121
